# Anti-Inflammatory Effects of Levalbuterol-Induced 11β-Hydroxysteroid Dehydrogenase Type 1 Activity in Airway Epithelial Cells

**DOI:** 10.3389/fendo.2014.00236

**Published:** 2015-01-12

**Authors:** Matthew J. Randall, Shannon F. Kostin, Edward J. Burgess, Laura R. Hoyt, Jennifer L. Ather, Lennart K. Lundblad, Matthew E. Poynter

**Affiliations:** ^1^Division of Pulmonary Disease and Critical Care Medicine, Department of Medicine, College of Medicine, The University of Vermont, Burlington, VT, USA

**Keywords:** albuterol, anti-inflammatory, 11beta-hydroxysteroid dehydrogenase, glucocorticoid, epithelium

## Abstract

Airway epithelial NF-κB activation is observed in asthmatic subjects and is a cause of airway inflammation in mouse models of allergic asthma. Combination therapy with inhaled short-acting β_2_-agonists and corticosteroids significantly improves lung function and reduces inflammation in asthmatic subjects. Corticosteroids operate through a number of mechanisms to potently inhibit NF-κB activity. Since β_2_-agonists can induce expression of 11β-HSD1, which converts inactive 11-keto corticosteroids into active 11-hydroxy corticosteroids, thereby potentiating the effects of endogenous glucocorticoids, we examined whether this mechanism is involved in the inhibition of NF-κB activation induced by the β-agonist albuterol in airway epithelial cells. Treatment of transformed murine Club cells (MTCC) with (R)-albuterol (levalbuterol), but not with (S)- or a mixture of (R + S)- (racemic) albuterol, augmented mRNA expression of *11*β*-HSD1*. MTCC were stably transfected with luciferase (luc) reporter constructs under transcriptional regulation by NF-κB (NF-κB/luc) or glucocorticoid response element (GRE/luc) consensus motifs. Stimulation of NF-κB/luc MTCC with lipopolysaccharide (LPS) or tumor necrosis factor-α (TNFα) induced luc activity, which was inhibited by pretreatment with (R)-, but not (S)- or racemic albuterol. Furthermore, pretreatment of GRE/luc MTCC with (R)-, but not with (S)- or racemic albuterol, augmented 11-keto corticosteroid (cortisone) induced luc activity, which was diminished by the 11β-HSD inhibitor glycyrrhetinic acid (18β-GA), indicating that there was a conversion of inactive 11-keto to active 11-hydroxy corticosteroids. LPS- and TNFα-induced NF-κB/luc activity was diminished in MTCC cells treated with a combination of cortisone and (R)-albuterol, an effect that was inhibited by 18β-GA. Finally, pretreatment of MTCC cells with the combination of cortisone and (R)-albuterol diminished LPS- and TNFα-induced pro-inflammatory cytokine production to an extent similar to that of dexamethasone. These results demonstrate that levalbuterol augments expression of *11*β-*HSD1* in airway epithelial cells, reducing LPS-induced NF-κB transcriptional activity and pro-inflammatory cytokine production through the conversion of inactive 11-keto corticosteroids into the active 11-hydroxy form in this cell type.

## Introduction

Asthma affects over 300 million Americans, causing morbidity and mortality through an inability to breathe effectively (1). The pathogenesis of asthma is characterized by airway inflammation, airflow obstruction, and bronchial hyperresponsiveness (2). A classic anti-inflammatory medication used in the treatment of asthma and in animal models of the disease is corticosteorids such as the endogenous human glucocorticoid, hydrocortisone, and synthetic glucocorticoids, including prednisone, budesonide, fluticasone, and dexamethasone (3), the latter of which have greater and protracted activity profiles compared to the endogenous molecules. This comes with a cost; long-term and high-dose glucocorticoid treatment can have serious side-effects, including a general feeling of malaise (e.g., weight gain and bloating), immunosuppression, cataracts, dysphonia, growth retardation in children, and osteoporosis in adults (4).

Glucocorticoids inhibit inflammation through multiple mechanisms (5), an important one involving interference with NF-κB, a pleiotropic transcription factor that is activated in response to inflammatory cytokines, mitogens, physical and oxidative stress, infection and microbial products, as well as allergens (6). NF-κB regulates a number of responses in mammalian cells, including the expression of many pro-inflammatory cytokines and chemokines (7, 8). NF-κB is active in the airway epithelium of both asthmatic patients (9–12) and in mouse models of the disease (13), wherein it is a signal critical in evoking pulmonary inflammation (8). We have demonstrated that inhibition of NF-κB activity in the airway epithelium of mice is sufficient to diminish many of the inflammatory features of both an ovalbumin (14) and a house dust mite (15) model of asthma, while activation of airway epithelial NF-κB exacerbates allergic airway disease (16) and enables allergic sensitization to an innocuous inhaled antigen (17).

The tissue levels of bioactive glucocorticoids are modulated by 11β-hydroxysteroid dehydrogenases (11β-HSD), which interconvert corticosteroids between inactive and active states (18); only the active forms of corticosteroids are capable of interacting with the glucocorticoid receptor (GR). 11β-HSD2 acts as a classical dehydrogenase by converting active 11-hydroxylated cortisol and corticosterone (in mice), into inactive 11-keto forms of cortisone and 11-dehydrocorticosterone, respectively (19, 20). 11β-HSD1, on the other hand, acts predominantly as an oxidoreductase, converting inactive 11-keto to active 11-hydroxylated corticosteroids (18, 21, 22). The activities of 11β-HSD enzymes at sites of glucocorticoid action, such as the airway epithelium, are essential for maintaining the balance between corticosteroid activity and inactivity, a process termed end-organ metabolism.

Another hallmark feature of asthma is hypersensitivity and hyperresponsiveness to bronchoconstricting stimuli, including inhalation of allergens, cold air, or methacholine. Agonists of the β_2_ adrenergic receptor (β_2_-agonists) such as albuterol increase intracellular cAMP, activate protein kinase A, induce transcription of responsive genes, and trigger relaxation of airway smooth muscle (23, 24). Consequently, β_2_-agonists are routinely employed as rescue medication for the treatment of acute asthma exacerbations (i.e., shortness of breath) (24). In addition, β_2_-agonists may also be prescribed as a maintenance medication, oftentimes in combination with corticosteroids, to keep airways open and prevent acute exacerbations.

The short-acting β_2_-agonist, albuterol, can be synthesized as two distinct enantiomers: (R)-(levalbuterol) and (S)-albuterol. A mixture of enantiomers, (R + S)-albuterol (racemic albuterol) is less costly to synthesize and is the most commonly used form of the drug (25). Although it has been debated whether there are pharmacological advantages to one or the other albuterol enantiomers (26), it is appreciated that levalbuterol is the “active” form conveying the therapeutic effect of albuterol (27–29), whereas (S)-albuterol has 100 times less affinity for the β_2_-receptor and may generate confounding effects (30, 31) or even function as an inhibitor for the effects of levalbuterol (32) when present in the racemic mixture.

Combination therapy with glucocorticoids and β_2_-agonists addresses two seemingly distinct hallmarks of the disease. However, in addition to their bronchodilatory effects, β_2_-agonists have anti-inflammatory effects on lung epithelium (33). The combination of corticosteroids and β_2_-agonists has been observed to decrease asthma symptoms, increase overall lung function, and inhibit inflammatory mediator production by airway epithelial and other pulmonary cell types better than either medication alone (34), which suggests that there are synergistic effects of the combination.

As corticosteroids in combination with β_2_-agonists generally provide asthma control (34, 35), yet protracted or systemic corticoid use can cause deleterious side-effects (4), the motivation for our study was to examine whether some of the anti-inflammatory effects of β_2_-agonists are mediated through their capacity to modulate the bioactivity of endogenous corticosteroids in a cell type and through a signaling pathway relevant to asthma pathogenesis. It has been demonstrated that the β-agonist, salbutamol, induces 11β-HSD1 oxidoreductase activity (conversion of inactive cortisone into active cortisol) in adipocytes (36). We explored in our studies whether a similar mechanism exists in airway epithelial cells, which express β-adrenergic receptors (37), and which can be exploited for therapeutic benefit in the treatment of asthma. Our long-term goal is to identify pathways that enable efficacious and affordable asthma control, without the systemic effects of oral corticosteroids.

## Materials and Methods

### Cell culture and treatment

Murine bronchiolar epithelial cells (MTCC, SV40 transformed Club cells) obtained from Dr. Francisco DeMayo (38) were cultured at 37°C in 95% humidified air containing 5% CO_2_ using DMEM (Gibco, Grand Island, NY, USA) containing 10% FBS (Cell Generation, Fort Collins, CO, USA), 2 mM L-glutamine, 50 U/ml penicillin, and 50 μg/ml streptomycin (Gibco). For experimentation, cells were seeded at 50,000 cells/cm^2^ and grown to confluence. Cells were treated with 10^−6^M (R)-, (S)-, racemic mixture of (R)- and (S)- albuterol (Sepracor, Marlborough, MA, USA), or 10 ng/ml TNFα (R&D Systems, Minneapolis, MN, USA) for 24 h after which RNA was isolated. Post-albuterol exposure, cells were treated with 100 ng/ml lipopolysaccharide (LPS; InvivoGen, San Diego, CA, USA) or 10 ng/ml TNFα for 16 h after which cell lysates were prepared for analysis of luciferase (luc) activity and cell-free conditioned media were collected for measurement of cytokines. 18β-glycyrrhetinic acid, an inhibitor of 11β-HSD, and dexamethasone, a synthetic form of cortisol, were purchased from Sigma (St. Louis, MO, USA).

### Gene expression

Total RNA was extracted from MTCC cultured on 12-well plates using the PrepEase RNA isolation kit (USM Corp., Cleveland, OH, USA) and reverse transcribed to cDNA using the iScript kit (Bio-Rad, Hercules, CA, USA). Real-time quantitative RT-PCR was performed using iQ Supermix (Bio-Rad) and intron-spanning primers on a Bio-Rad Chromo4. Primers were designed for mouse *Hsd11b1* (5′-TTA TTG TCA AGG CGG GAA A5-3′ and 5′-GGC GTC AAT TAT CCC AGA GA-3′), *Hsd11b2* (5′-TCA TCA CCG GTT GTG ACA CT-3′ and 5′-GGT ATG GCA TGT CTC CTG CT-3′), and *Gapdh* (5′-ACG ACC CCT TCA TTG ACC TC-3′ and 5′-TTC ACA CCC ACT ACA AAC AT-3′). The levels of gene expression were normalized to *Gapdh* levels and relative expression was calculated according to the comparative cycle threshold (ΔΔC_T_) method, as previously described (17).

### Luciferase assay

MTCC were stably transfected with a mammalian expression vector containing an NF-κB-regulated luc reporter (Biomyx Technology, San Diego, CA, USA) or a glucocorticoid response element (GRE; from the mouse mammary tumor virus long terminal repeat) regulated luc reporter [pHH-luc (39) from Nordeen, University of Colorado Health Sciences Center via Daynes, University of Utah) using lipofectamine (Life Technologies, Grand Island, NY, USA). Stable transfectants were selected using neomycin or hygromycin (Sigma). For quantitation of luc activity, equal quantities of cellular protein, quantitated by the Bradford method (Bio-Rad), were extracted following treatment and analyzed using a Berthold Lumat LB9501 luminometer and luciferin substrate, as previously described (13).

### Cytokine measurement

Bio-Plex (Bio-Rad, Hercules, CA, USA) kits were designed containing coupled polystyrene beads and antibodies recognizing mouse IL-1β, IL-6, IL-12p40, IL-12p70, eotaxin, GM-CSF, G-CSF, KC, MCP-1, MIP-1α, MIP-1β, and RANTES. All assays were performed in duplicate according to manufacturer’s instructions. Briefly, 50 μl of coupled beads were added to each well of a pre-wet 96-well microtiter plate with filter bottoms, washed twice using a Bio-Rad (Hercules, CA, USA) Bio-Plex Pro II wash station with the vacuum plate carrier, and 100 μl of sample (cell-free conditioned media), standard, or assay buffer (background) was added to each well. The plates were covered, shaken vigorously for 1 min on an IKA (Wilmington, NC, USA) MTS 2/4 digital microtiter plate shaker and then moderately shaken for 4 h at room temperature. After washing, 25 μl of biotinylated detection antibodies were added to each well for 1 h with shaking followed by addition of 50 μl of streptavidin-PE to all wells for 30 min with shaking. The wells were washed and the beads were resuspended in 125 μl sheath fluid and shaken to resuspend. Data were acquired at low PMT setting using the Bio-Rad Bio-Plex suspension array system and Bio-Plex Manager 6.0 software. Fluorescence intensity of the background was subtracted from the values for each sample and standard for each specific bead. Seven-point standard curves were generated from fourfold dilutions of standards provided in the Bio-Plex kits, which were analyzed using five-place logistic regression from standards within 95–105% of the expected values. Upper levels of quantitation and lower levels of quantitation were calculated by the Bio-Plex Manager 6.0 software. Reported concentrations are in picograms per milliliter of the cytokines that were induced by LPS and TNFα and were inhibited by dexamethasone (IL-6, GM-CSF, G-CSF, KC, MCP-1, MIP-1α, and RANTES).

### Statistical calculations

Data are presented as mean ± SEM and were analyzed by two-way ANOVA followed by inter-groups analysis by two-tailed unpaired *t*-test with Bonferroni correction for multiple comparisons using GraphPad Prism 6 (San Diego, CA, USA). A *p* value <0.05 was considered statistically significant.

## Results

### R-albuterol induces 11β-HSD1 gene expression

**Figure 1:**
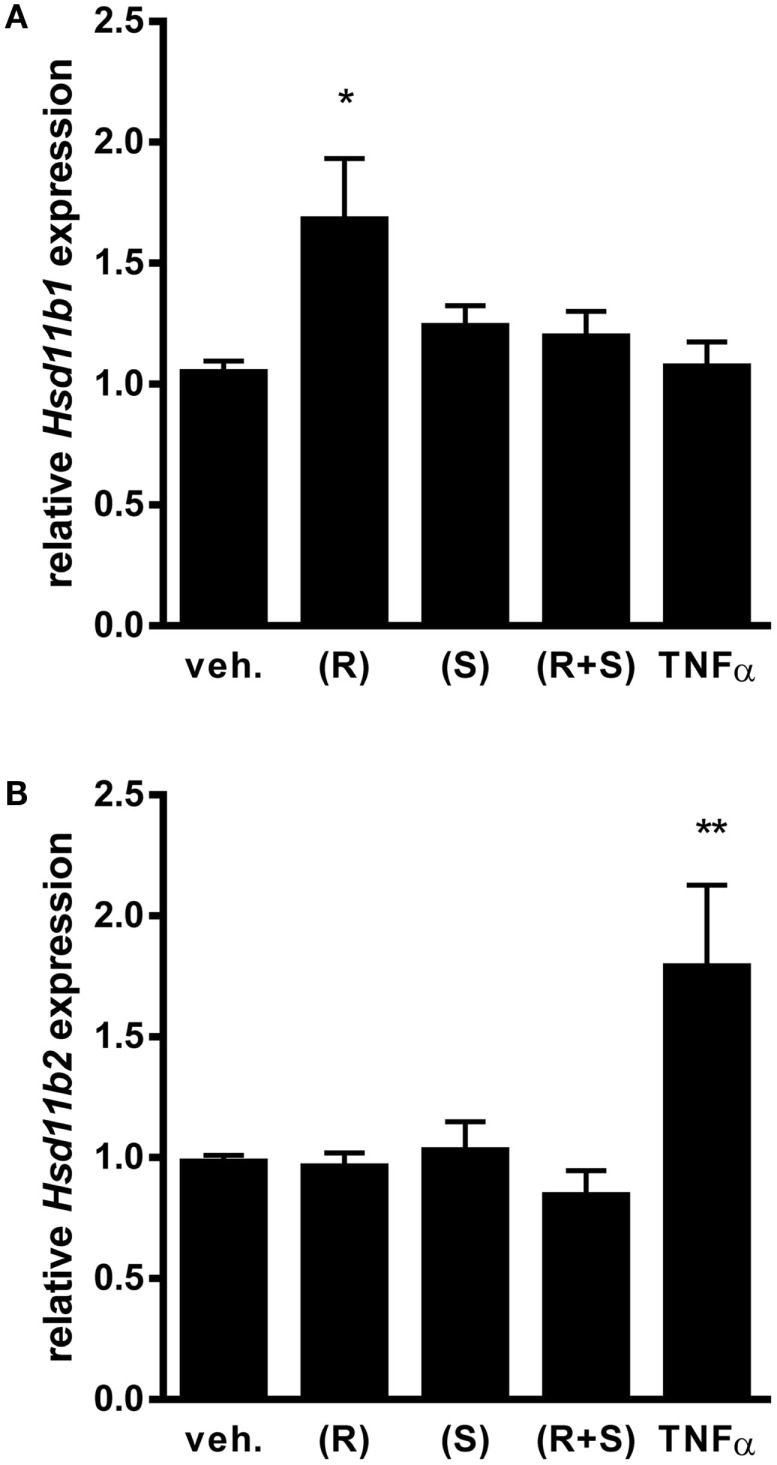
**Exposure to (R)-albuterol, but not (S)-albuterol or racemic (R + S)-albuterol, induces mRNA expression of 11β-HSD1, but not 11β-HSD2, in airway epithelial cells**. MTCC were exposed to 10^−6^M (R)-albuterol (R), (S)-albuterol (S), racemic (R + S)-albuterol, or 10 ng/ml TNFα. Twenty-four hours later, RNA was isolated, cDNA was generated, and 11β-HSD1 (Hsd11b1), 11β-HSD2 (Hsd11b2), and GAPDH (Gapdh) gene expression were analyzed by quantitative PCR. Relative expression of 11β-HSD1 **(A)** and 11β-HSD2 **(B)** were calculated. Data were pooled from three separate experiments. *n* = 6–9/group; *p ≤ 0.05 and **p ≤ 0.01 compared to vehicle.

For our studies, we used a transformed non-ciliated murine airway epithelial cell line, MTCC (38). These cells, also referred to as Club cells, represent an abundant cell type lining the conducting airways of mice and humans, which are amongst those cells anatomically positioned *in vivo* such that they are exposed to inhaled bronchodilators and steroids (40). Furthermore, we have reported that murine Club cells are an important regulator of pro-inflammatory cytokine production through their capacity to activate the transcription factor NF-κB (13–15, 17). For the studies conducted herein, we first confirmed by Western blot that the MTCC, like the mouse lung, do indeed express the β_2_-adrenergic receptor (data not shown). Although not as abundant on a per microgram protein basis as the whole lung, expression of β_2_-adrenergic receptor protein suggested that these cells were well-suited to the investigation of the effects of albuterol on airway epithelium. We next investigated whether MTCC express mRNA for 11β-HSD isoforms, *11*β*-HSD1*, and *11*β*-HSD2*, and whether 11β-HSD expression was affected by albuterol treatment. We exposed MTCC to (R)-albuterol, (S)-albuterol, or (R + S) albuterol at a number of doses and for a number of durations (data not shown) in order to establish relevant exposure regimens for our studies. When MTCC were exposed to 10^−6^M (R)-albuterol for 24 h there was a significant increase in the expression of *11*β*-HSD1* (Figure 1A). However, neither (S)-albuterol nor (R + S)-albuterol influenced *11*β*-HSD1* mRNA expression. In addition, none of the albuterol enantiomers affected the expression of *11*β*-HSD2* (Figure 1B). As a positive control, 24 h exposure to 10 ng/ml recombinant mouse TNFα significantly increased *11*β*-HSD2* expression. These results demonstrate that albuterol, in particular (R)-albuterol, selectively induces *11*β*-HSD1* expression in airway epithelial cells.

### LPS- and TNFα-induced NF-κB activity is diminished by (R)-albuterol treatment

**Figure 2:**
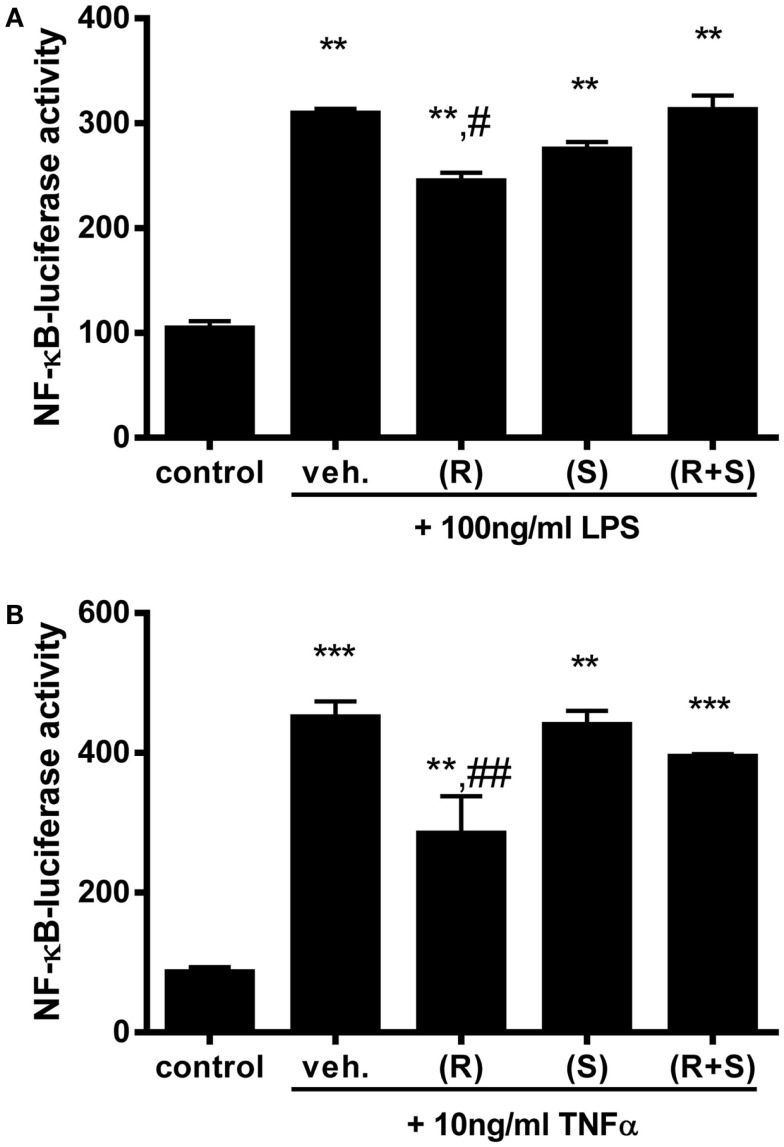
**Pre-exposure to (R)-albuterol reduces LPS- and TNFα-induced NF-κB activity in airway epithelial cells**. NF-κB-luciferase MTCC were exposed to vehicle (veh.) or 10^−6^M (R)-albuterol (R), (S)-albuterol (S), or (R + S)-albuterol for 24 h. All cells except control were then exposed to 100 ng/ml LPS **(A)** or 10 ng/ml TNFα **(B)**. Sixteen hours later, cell lysates were prepared, luciferase activity was measured, and protein concentration was determined. *n* = 5–6/group and the experiment was repeated twice; **p ≤ 0.01 compared to control, ***p ≤ 0.001 compared to control, ^#^*p* ≤ 0.05 compared to vehicle, ^##^*p* ≤ 0.01 compared to vehicle.

Since R-albuterol exposure significantly increased *11*β*-HSD1* expression in MTCC, we investigated whether albuterol would diminish NF-κB activity induced by two prototypical agonists, LPS and TNFα. LPS is an abundant environmental agonist of TLR4, a receptor important for cellular responses to several asthma-relevant allergens (41, 42). TNFα is elevated in allergic asthma and in mouse models of allergic airway disease, wherein it initiates and amplifies pulmonary inflammatory responses (43). Having established the half-maximal doses of these agonists, MTCC stably transfected with a NF-κB-dependent luc reporter gene were exposed to 100 ng/ml LPS or 10 ng/ml TNFα in the absence or presence of 10^−6^M (R)-albuterol, (S)-albuterol, or (R + S)-albuterol. While LPS significantly induced NF-κB-luc activity, simultaneous exposure with albuterol did not diminish LPS-induced NF-κB activity (data not shown). Similar results were measured when 10 ng/ml TNFα was substituted for LPS (data not shown). Although we had hypothesized that simultaneous treatment with albuterol would diminish LPS- or TNFα-induced NF-κB activation, we tested whether pretreatment with albuterol would be more effective. Therefore, MTCC were left untreated or were treated with 10^−6^M (R)-, (S)-, or (R + S)-albuterol for 24 h. Cells were then treated with 100 ng/ml LPS or 10 ng/ml TNFα for 16 h and analyzed for NF-κB activity. While treatment with LPS (without or with albuterols) significantly increased NF-κB-dependent luc reporter activity, only pretreatment with 10^−6^M (R)-albuterol significantly reduced LPS-induced NF-κB activity (Figure 2A). Similarly, pretreatment with 10^−6^M (R)-albuterol also significantly reduced TNFα-induced NF-κB activity (Figure 2B).

### Suppression of NF-κB activity by (R)-albuterol is dependent upon 11β-HSD1

**Figure 3:**
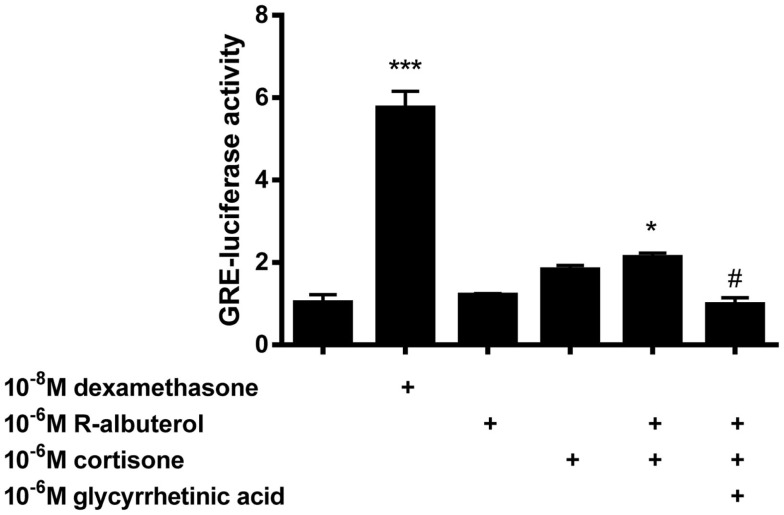
**Pre-exposure of airway epithelial cells to (R)-albuterol for 24 h, followed by cortisone exposure for 16 h, augments GRE-luciferase activity, which requires the activity of 11β-HSD**. GRE-luciferase MTCC were exposed to 10^−8^M dexamethasone or 10^−6^M (R)-albuterol. Twenty-four hours later, 10^−6^M cortisone alone or with 10^−6^M glycyrrhetinic acid was added for 16 h and luciferase activity and total protein were measured. *n* = 5–6 samples/group and the experiment was repeated twice; ***p ≤ 0.001 compared to untreated, *p ≤ 0.05 compared to untreated, ^#^*p* ≤ 0.05 compared to (R)-albuterol + cortisone.

To elucidate whether the capacity of (R)-albuterol treatment to diminish NF-κB activity was partially due to the activity of glucocorticoids, GRE-dependent luc reporter transfected MTCC were used. Since we had demonstrated that 24 h pre-exposure of MTCC to (R)-albuterol augmented 11β-HSD1 expression and diminished LPS- and TNFα-induced NF-κB activity, GRE-luc MTCC were either left unexposed or exposed to 10^−6^M (R)-albuterol for 24 h. Post-albuterol exposure, 10^−6^M cortisone (with or without 10^−6^M of the 11β-HSD inhibitor glycyrrhetinic acid) was then administered to the cell culture medium and incubated for an additional 16 h. As a positive control, GRE-luc MTCC were treated with 10^−8^M of the synthetic, 11-hydroxy (bioactive) glucocorticoid, dexamethasone for the entire 30 h of the experiment. Pretreatment with (R)-albuterol followed by exposure to cortisone modestly but significantly induced GRE-luc activity (Figure 3). The effect of (R)-albuterol combined with cortisone was dependent on the activity of 11β-HSD1, as the addition of 10^−6^M of the 11β-HSD inhibitor glycyrrhetinic acid significantly diminished GRE-luc activity.

**Figure 4:**
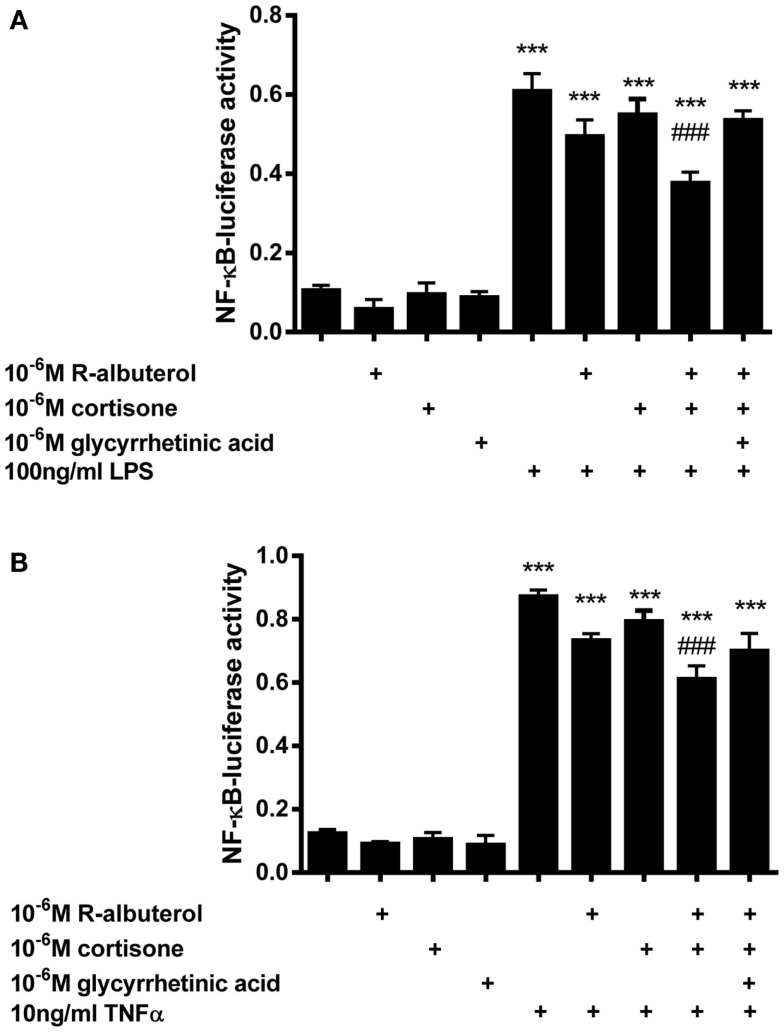
**Pre-exposure of MTCC to (R)-albuterol and cortisone for 24 h, followed by exposure to LPS or TNFα for 16 h, diminishes NF-κB-luciferase activity, which requires the activity of 11β-HSD**. NF-κB-luciferase MTCC were exposed for 24 h to 10^−6^M cortisone alone or to 10^−6^M (R)-albuterol with or without 10^−6^M cortisone and 10^−6^M glycyrrhetinic acid. Twenty-four hours later, 100 ng/ml LPS **(A)** or 10 ng/ml TNFα **(B)** were added to the cell culture medium. Cells were then lysed 16 h later using reporter lysis buffer. Luciferase activity and total protein were then measured. *n* = 4–6 samples/group and the experiment was repeated twice; ***p ≤ 0.001 compared to untreated, ^###^*p* ≤ 0.001 compared to LPS **(A)** or TNFα **(B)**, ^#^*p* ≤ 0.05 compared to LPS **(A)** or TNFα**(B)**.

Subsequently, we assessed the role of 11β-HSD1 in albuterol suppression of NF-κB activity using NF-κB-luc MTCC. Cells were exposed for 24 h to 10^−6^M cortisone alone or to 10^−6^M (R)-albuterol with or without 10^−6^M cortisone and 10^−6^M glycyrrhetinic acid. Following this exposure, cells were stimulated with 100 ng/ml LPS or 10 ng/ml TNFα for 16 h and NF-κB-dependent luc activity was measured. As is shown in Figure 4A, only the combined pretreatment of (R)-albuterol and cortisone significantly, albeit modestly, decreased LPS-induced NF-κB-luc activation. Similarly, as is shown in Figure 4B, the combined pretreatment with (R)-albuterol and cortisone significantly, albeit modestly, decreased TNFα-induced NF-κB-luc activation. Pretreatment with the inactive glucocorticoid, cortisone, had no effect on LPS- or TNFα-induced NF-κB-luc activity in the absence of (R)-albuterol. In the cases of both LPS and TNFα exposure, pretreatment with the 11β-HSD inhibitor, glycyrrhetinic acid, prevented much of the effect of combined pretreatment with (R)-albuterol and cortisone, implicating the importance of 11β-HSD1 activity. These experiments demonstrate that only (R)-albuterol augmented 11-keto corticosteroid induced GRE-luc activation, indicating that there was a modest but statistically significant conversion of inactive 11-keto to active 11-hydroxy corticosteroids capable of inhibiting LPS- and TNFα-induced NF-κB activity.

### (R)-albuterol suppresses production of NF-κB-regulated cytokines

**Figure 5:**
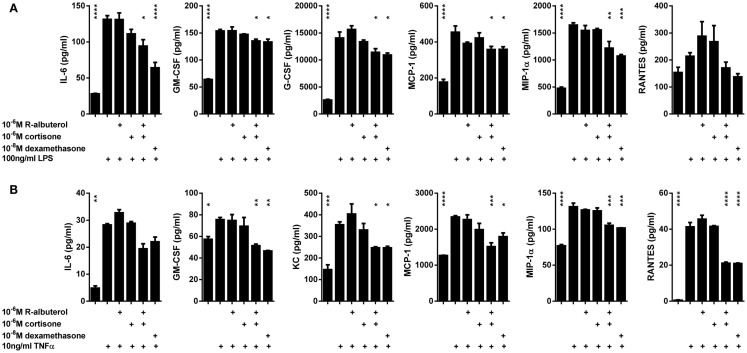
**Pre-exposure of MTCC to (R)-albuterol and cortisone diminishes LPS- and TNFα-induced pro-inflammatory cytokine production**. MTCC were exposed to 10^−6^M (R)-albuterol, with or without 10^−6^M cortisone or 10^−6^M glycyrrhetinic acid, or to 10^−8^M dexamethasone. Twenty-four hours later, 100 ng/ml LPS **(A)** or 10 ng/ml TNFα **(B)** were added to the cell culture medium. Cell-free conditioned media were then collected 16 h later and pro-inflammatory cytokine levels were measured. *n* = 3 samples/group and the experiment was repeated twice; *p ≤ 0.05, **p ≤ 0.01, ***p ≤ 0.001 compared to LPS **(A)** or TNFα **(B)**.

We have previously reported the critical contribution that NF-κB activation in non-ciliated airway epithelial cells (Club cells) has in mouse models of acute lung injury (44) and allergic airway disease (13–17), wherein these cells secrete cytokines that orchestrate innate and adaptive immune responses. Using MTCC cells as an *in vitro* model, we left the cells untreated or pretreated the cells for 24 h with 10^−8^M dexamethasone, 10^−6^M cortisone, 10^−6^M (R)-albuterol, or a combination of 10^−6^M (R)-albuterol and 10^−6^M cortisone. Cells were then stimulated with 100 ng/ml LPS or 10 ng/ml TNFα for 16 h and cytokines were measured from cell-free conditioned medium. As is shown in Figure 5A, only the combined pretreatment of (R)-albuterol and cortisone significantly decreased LPS-induced production of IL-6, GM-CSF, G-CSF, MCP-1, and MIP-1α. Similarly, the combined pretreatment with (R)-albuterol and cortisone significantly decreased TNFα-induced production of IL-6, GM-CSF, KC, MCP-1, MIP-1α, and RANTES (Figure 5B). Notably, pretreatment with cortisone alone had no effect on LPS- or TNFα-induced cytokine production in the absence of (R)-albuterol. These experiments implicate (R)-albuterol-regulated 11β-HSD1 activity as a potentially important modulator of pro-inflammatory signaling and cytokine production in airway epithelial cells.

## Discussion

The combination therapy of corticosteroids and β_2_-agonists has been typically demonstrated as an effective therapy for many patients with asthma and COPD. These drugs target airflow obstruction and airway inflammation, two of the most pressing features of these pulmonary diseases. β_2_-adrenergic and GRs are expressed by many cells within the lung, including airway epithelium, one of the principle cell types exposed when these drugs are delivered by inhalation. While many of the cell signaling events activated following β-agonist or glucocorticoid exposure have been elucidated, there remains a gap in our understanding of how the two drugs may interact synergistically to provide better disease control than each alone. One possibility is that β_2_-agonists induce changes in gene expression that allow for differential sensitivity to endogenous glucocorticoids. Thereby, higher local concentrations of bioactive glucocorticoids may be able to provide exceptional anti-inflammatory activity at the sites in which that activity is most needed, without the potentially deleterious side-effects that can be induced by systemic steroids. In support of this hypothesis, GR function has been reported to increase following β_2_-agonist treatment (35).

11β-HSD enzymes interconvert inactive circulating 11-keto glucocorticoids into bioactive 11-hydroxy steroids capable of interacting with the GR (45). We have demonstrated that (R)-albuterol significantly reduces both LPS- and TNFα-induced NF-κB activity while increasing GRE activation in an 11β-HSD1 dependent manner, manifesting in lower levels of pro-inflammatory cytokine production from a transformed mouse airway epithelial cell line. In this study, we first compared levalbuterol to both (S)- and racemic albuterol, wherein we identified that levalbuterol moderately, but selectively, induces expression of *11*β*-HSD1* but not *11*β*-HSD2*, whereas (S)- and racemic albuterol had no effect on either. The comparison of (R)-, (S)-, and (R + S)-albuterol has been studied extensively in clinical or basic research, with several outcomes supporting the use of (R)- over (S)-albuterol (28, 46–48). In this vein, there is evidence to suggest that the (R)-enantiomer is the bioactive component and that the S-enantiomer may even have detrimental effects (49). Although others have stated that there is an opposite effect or no difference between enantiomer treatments (26, 50), one could argue that the complexity of asthma as a disease could potentiate such differences in treatment efficacy. It is not clear why in our studies neither the (S)- nor racemic (R + S)-albuterol induced 11β-HSD1 expression. One potential explanation for the lack of response from albuterols containing the (S)-enantiomer lies in the higher binding affinity of (R)-albuterol to the β_2_-adrenergic receptor (32). While our studies did not enable us to explore this possibility in our airway epithelial cell model system, the data clearly demonstrate the ability of only (R)-albuterol to elicit anti-inflammatory effects.

We further addressed whether the anti-inflammatory property of levalbuterol requires endogenous glucocorticoid signaling. Previous studies that have attempted to distinguish a route through which albuterol enhances corticosteroid sensitivity primarily focus on downstream signaling of the GR following glucocorticoid exposure and neglect to consider that albuterol may modulate levels of localized endogenous corticosteroid. A study by Usmani et al. identified that long-acting β_2_-agonists could increase nuclear localization of the GR and increase DNA binding of GR enhancing glucocorticoid function (51). Our data are complimentary in this regard for levalbuterol, a short-acting β_2_-agonist, since we observed an enhanced GRE-luc activity following levalbuterol treatment, although only in the presence of cortisone. Conversely, Eickelberg et al. have suggested that long-acting β_2_-agonists can activate the GR independently of corticosteroid binding (52) whereas Loven et al. suggest a GR independent mechanism (53). Albeit, we attribute the levalbuterol-mediated GRE-luc activity observed to GR activation via the conversion of cortisone to cortisol by 11β-HSD1 given that we can inhibit the GRE activity by the potent 11β-HSD inhibitor, 18β-GA. The levalbuterol-mediated suppression of NF-κB activity following TNFα or LPS stimulation was also partially prevented following the administration of 18β-GA indicating a role for 11β-HSD1 in the anti-inflammatory function of levalbuterol. Furthermore, the capacity of 18β-GA to partially block the inhibitory effects of (R)-albuterol on GRE- and NF-κB-dependent luc activity specifically implicates necessity for the oxidoreductase (11-keto to 11-hydroxy conversion) activity of 11β-HSD1 for albuterol-mediated suppression of NF-κB. By extension, the requirement for (R)-albuterol pretreatment and the addition of cortisone for the diminution of LPS- and TNFα-induced pro-inflammatory cytokine production implies that active glucocorticoids mediate this effect.

Whether the effects of (R)-albuterol observed *in vitro* would translate into biologically significant reductions in inflammatory cytokine or chemokine production *in vivo* remain unknown. In addition, it is uncertain whether airway epithelium is the most relevant cell type in which to study 11β-HSD activities in response to β_2_-agonists. While the MTCC cell line was utilized for our studies based on the important functions we have attributed to NF-κB activity in Club cells of mice (13–17, 44), MTCC retain only a limited number of the characteristics of *in vivo* Club cells (38) and are not representative of the more abundant ciliated airway epithelial cells lining the conducting airways of humans. Furthermore, our cell model system did not involve culturing at an air-liquid interface, which is more representative of the *in vivo* state of differentiated airway epithelium. Our studies should be extended to primary cells from healthy mice and those with allergic airway disease, as well as to healthy human subjects and those with different asthma endotypes (54). In addition to epithelia, many other types of cells present in the inflamed airway, including lymphocytes and macrophages that are pathogenic in asthma and COPD, express 11β-HSD1 (55, 56) and may be modulated in their activity by β_2_-agonists (57, 58). Nevertheless, the modest reductions we observed *in vitro* are of a similar magnitude to what has been recently reported from an elegant *in vivo* study using a mouse model of allergic asthma in which (R)-albuterol modestly diminished inflammation and NF-κB activity (58). Finally, whether the observed effects of (R)-albuterol would also be mimicked or even enhanced by a long-acting β_2_-agonists, such as (R,R)-formoterol, which do diminish inflammatory cytokine production by human airway epithelial cells *in vitro* (59), remains an intriguing question. Certainly, there is the potential that long-term augmentation of airway epithelial 11β-HSD1 oxidoreductase activity could provide endogenous glucocorticoids with powerful and localized anti-inflammatory activities. Our finding that pretreatment of epithelial cells with (R)-albuterol was required to elicit inhibitory effects on LPS- or TNFα-induced activation suggests that maintenance therapy [with (R)-albuterol] may be more beneficial than as-needed treatment.

**Figure 6:**
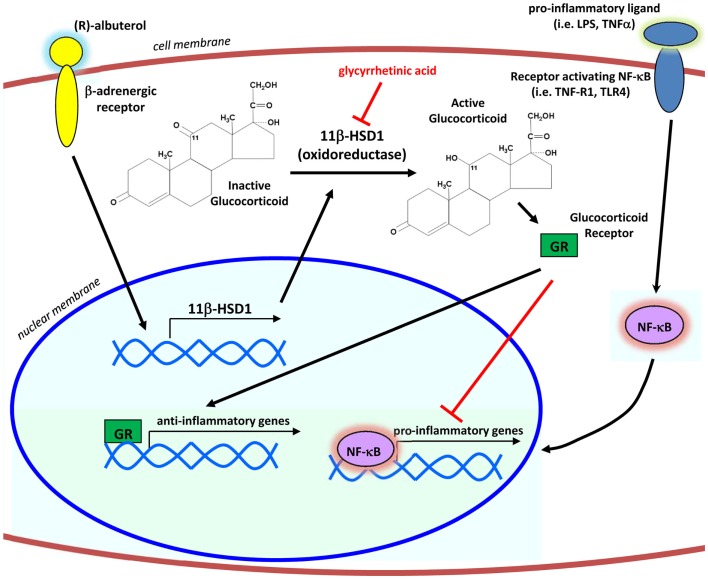
**Proposed mechanism of action**. By signaling through the β2-adrenergic receptor, (R)-albuterol transcriptionally upregulates the mRNA expression and oxidoreductase activity of 11β-HSD1 in airway epithelial cells, thereby potentiating the anti-inflammatory effects of endogenous glucocorticoids to inhibit activity of the pro-inflammatory transcription factor NF-κB.

Taken together, our results define a mechanism by which levalbuterol may suppress airway inflammation (Figure 6). Translation of our *in vitro* findings suggests that β_2_-agonist induced enhancement of 11β-HSD1 oxidoreductase activity in the airway epithelium of asthmatic patients has the potential to increase the metabolic activation of their endogenous cortisol and suppress baseline inflammatory responses, in particular those involving transcriptional control by NF-κB. Through the activation of endogenous corticosteroids selectively in the affected lung epithelium, levalbuterol and potentially other β_2_-agonists could be used as a tool enabling clinicians to circumvent the use of long-term or systemic corticosteroid therapies for the treatment of inflammatory airway diseases.

## Conflict of Interest Statement

The authors declare that the research was conducted in the absence of any commercial or financial relationships that could be construed as a potential conflict of interest.
